# Determinants of stock-outs of first line anti-tuberculosis drugs: the case of public health facilities of Addis Ababa city administration health bureau, Addis Ababa, Ethiopia

**DOI:** 10.1186/s12913-022-08430-3

**Published:** 2022-08-17

**Authors:** Kassech Sintayehu, Eden Dagnachew Zeleke, Busha Temesgen, Meron Kifle, Dawit Getachew Assefa, Kibebew Zenebe, Ashenafi Kassahun, Ben Tegegn Yimer

**Affiliations:** 1 KNCV (Koninklijke Nederlandse Chemische Vereniging (Royal Dutch Chemical Association) Tuberculosis Foundation, Addis Ababa, Ethiopia; 2 Ethiopian food and drug Authority, Addis Ababa, Ethiopia; 3 Department of Midwifery, Institute of Health, Bule Hora University, P.O.BOX-22497/1000, Bule Hora, Ethiopia; 4 Department of logistics and supply chain management, School of commerce, Addis Ababa University, Addis Ababa, Ethiopia; 5 Department of Nursing, College of health science, Dilla University, Dilla, Ethiopia; 6 National Blood Bank Services, Addis Ababa, Ethiopia; 7 Ethiopian Pharmaceuticals Supply Agency, Addis Ababa, Ethiopia; 8 Addis Ababa City Administration Health Bureau, Addis Ababa, Ethiopia

**Keywords:** Stock out, Anti TB drugs, Health system support, Performance of the supplying hub, Public health facilities, Addis Ababa

## Abstract

**Background:**

The health sectors success has been determined by consistent and reasonably priced health commodities supply. Despite possible death from the disease, Tuberculosis (TB) can be prevented with early diagnosis and appropriate treatment for which enough, effective, and qualified medicines need to be available. However, studies revealed stock of anti-TB drugs in health facilities. Here we present the recent finding on determinants of stock out of Anti-TB drug at public health facilities of Addis Ababa.

**Objective:**

This study aimed to identify determinants of stock outs of first line anti TB drugs at public health facilities under Addis Ababa City Administration Health Bureau.

**Method:**

Mixed study design were employed. A total of 106 facilities were included in the sampling frame and data were collected from the study population such as drug store managers of health facilities providing TB treatment using semi structured questionnaire and through in-depth interview with Addis Ababa hubs of the Ethiopian Pharmaceuticals Supply Agency (EPSA), Addis Ababa City Administration Health Bureau and selected heads of pharmacy departments of health facilities from May 1–30, 2020 considering one year back retrospective data from March 20,2019 to March 20,2020. Structured record review of data from Logistics Management Information System (LMIS) tools having TB drugs was done using structured observation checklist. Data were entered, cleaned, and analyzed using SPSS Version 20. Both descriptive and multiple logistic regression analysis were performed.

**Result:**

52(62.7%) of health facilities encountered stock out for at least one of these drugs during the past 1 year. Rifampicin *75* mg + Isoniazid *50 mg* (RH 75/50 mg) were most stocked out first line anti-TB drug from 33(39.8%) of facilities with 17 mean stocks out days while Rifampicin *75* mg + Isoniazid *50 mg* + Pyrazinamide 150 mg (RHZ 75/50/150 mg) were the least first line anti-TB drug stocked out from facilities with mean 5 days of stock out. Delayed supply of anti TB drug from EPSA, delivery of reduced quantity of anti TB drugs by EPSA and stocked out of anti TB Drugs at EPSA were significant determinate factors of stock out of first line anti-TB drug from facilities with 95%CI of 10.34(2.167–49.329), 11.452(2.183–60.079) and 5.646(1.240–25.707) respectively.

**Conclusion:**

Above median of health facilities encountered stock out of first line anti-TB drug in Addis Ababa. Delayed supply of anti TB drug from EPSA, delivery of reduced quantity of anti TB drugs by EPSA and stocked out of anti TB Drugs at EPSA were significant determinate factor of stocked out of first line anti-TB drug from facilities. EPSA and other responsible bodies shall work collaboratively to improve their service and ensure availability of adequate amount of Anti TB drug in health facilities.

**Supplementary Information:**

The online version contains supplementary material available at 10.1186/s12913-022-08430-3.

## Background

Tuberculosis (TB) have been among the top 10 causes of mortality. Globally, 10 million peoples have got the disease in which 151 new TB cases per 100,000 population reported in 2018 and Ethiopia is among the 30 high TB burden countries [1].

As per the Ethiopian Food and Drug Authority (EFDA), “ safe, effective, and affordable medicines of the required and assured quality, in adequate quantity should always be available for the provision of proper healthcare service ” [2].

The consequence of medicines stock out from health facilities has been reported by different studies. Such as discontinuation of treatment by patients, hopelessness in the health systems, extra cost, discouraging the clinicians , care givers, and providers [3, 4] high risk for drug resistance and adverse effects [4, 5] and subsequently, put many patients on higher risk of morbidity and mortality and confronting the public health [5, 6] and decrease TB cure rate and treatment success rate [7].

Previous study in 2016 showed > 80% availability of key medicine for infectious diseases in Ethiopia’s public health facilities [8] and recently conducted study on service readiness assessment shows <50% availability of most essential drugs [9]. A study at public health facilities of Addis Ababa showed availability of Anti TB drugs on the day of visit were 72.5% and 68% at the hospital and health center, respectively [10]. Most deaths from TB could be prevented with rapid diagnosis and appropriate treatment for which sufficient amount of effective and qualified medicines is required , hence TB control program should be conscious about the availability of Anti-TB drugs for proper implementation [11].

Studies determined different reasons for Stock-outs such as poor communication and coordination among stakeholders, poor inventory management practice [12] educational level of stock managers, poor practice of use of ICT by stock managers [13] poor availability of transportation and road infrastructure [4, 14] unexpected demand changes or fluctuations, limited health budgets, raw material problems, frequently changing regulatory requirements [15] and, supply of short expiry drugs from a supplying warehouse [16].

There has been contrariety between reports about the availability of infectious disease medicines and Addis Ababa city Administration health bureau report on stock out for TB medicines in public health facilities providing TB treatment services. Therefore, this study aimed to investigate the magnitude and determinant factors for stock out of first line anti TB drugs at public health facilities under Addis Ababa City Administration Health Bureau.

## Methods and materials

### Study design

Mixed design (quantitative and qualitative) were employed. In-depth interview and secondary data review was done.

### Study area and period

Addis Ababa city is administratively subdivided into 10 sub-cities and has a total population of 3686, 068 million. From 104 public health facilities (98 health center and 6 public hospitals), all health centers and 4 hospitals were providing TB diagnostic and treatment services. The study period was from March 20,2019 to March 20,2020 and data collection time was from May 1–30, 2020. In this study retrospective collection of data on stock out situations in the previous one-year was done at the same time collecting data on determinants for stock-outs.

### Source population

All public health facilities of Addis Ababa providing tuberculosis diagnosis and treatment services which totally were 102 health facilities, 2 Regional Health Bureau, 2 supplying pharmaceutical warehouses (EPSA hubs).

### Study population

Drug store manager and heads of pharmacy department from public health facilities, TB/HIV and pharmaceutical logistics case team from regional health bureau and program drugs distribution coordinator from the supplying pharmaceutical warehouses (EPSA hubs).

### Eligibility criteria

#### Inclusion criteria

- ➢ Being head and store manager of the selected facility.
- ➢ The respondent should work on this position at least for 6 months.

### Study variables

#### Dependent variable

- ✓ Anti-TB first line drug Stock out

#### Independent variable

- ✓ ICT
- ✓ Health system support
- ✓ Performance of supplying hub

### Sample size and sampling method

A total of 106 respondents were targeted to be interviewed from 102 health facilities, regional health bureau and 2 supplying hubs. Census and non-probability sampling methods was applied to include all the eligible public health facilities providing TB treatment services in Addis Ababa. Study participants were selected based on the year of experience and their knowledge towards the topic.

### Data collection procedure and tool

#### Quantitative data collection methods

Semi-structured questionnaire was administered to drug store managers at 102 health facilities and structured record review of data from Logistics Management Information System (LMIS) tools and bin card having TB drugs was done using structured observation checklist to extract stock out data for the for previous 1 year.

#### Qualitative data collection methods

Using in-depth interview with totally 5 heads of pharmacy departments, 2 program drugs distribution coordinators and 2 TB/HIV and pharmaceutical logistics case team. All the interview session were recorded, and personal identifiers were deidentified for the analysis.

The data collection questions for primary source and checklists for secondary data sources was adapted from tools of other researchers and Logistics System Assessment Tool (LSAT) respectively and indicators to measure stock-outs was utilized from Logistics Indicators Assessment Tool (LIAT) and tools developed by the pharmaceuticals fund and supply agency [17, 18].

### Data quality assurance

Data collectors were trained by the principal investigator before the data collection initiated. A structured questionnaire and observational checklist were used to avoid bias. The tool was piloted and revised based on the finding accordingly. Data were checked for completeness on daily bases.

### Data analysis

Data were entered, cleaned, and analyzed using SPSS Version 20. Both descriptive and multiple logistic regression analysis were performed. Assumptions were checked before regression analysis and p value less than 0.05 were considered statistically significant. Thematic analyses were performed for qualitative data analysis. Qualitative data was handled manually and categorized and individually coded with themes related to the variables and added as an explanation for the findings of quantitative analysis.

## Result

It was planned to collect the data from all TB treatment providing facilities (102), however due to the COVID 19 pandemic, 14 of them were turned in to isolation center. The response rate was 83/88 (94.3%). From those participants, 40(48.2%) and 43(51.8%) were female and male respectively with mean (±SD) age of 31(±5.51). Majority of the respondents 48 (57.8 %) hold a bachelor’s degree, 33 (39.8 %) diploma and 2(2.4%) hold postgraduate degree. 46(55.42%) 0f participant had (0-5 years of experience and 37(44.58%) had 6-13years of experience. Two participants from supply hub and two from regional health bureau were interviewed about determinant factor of stock out of first line Anti-TB drug.

### Stock out rate of first line anti-tuberculosis drugs

The result revealed that 52(62.7%) of health facilities encountered stock out for at least one of the first line anti TB drugs during the past 1 year. As shown in Table 1 RH 75/50mg (Rifampicin 75mg + Isoniazid 50mg) was most stocked out first line anti-TB drug from 33(39.8%) of facilities with 17 mean stocks out days while RHZ 75/50/150mg (Rifampicin 75mg + Isoniazid 50mg + Pyrazinamide 150mg) was the least first line anti-TB drug stocked out from facilities with mean 5 days of stock out.

**Table 1:** Findings from Health Facilities on Stock out for each of first line anti TB Drugs as of March 20/2019 to March 20/2020

First Line Anti TB drugs	Facilities stock Out n (%)	# Of Days of Stock Out Mean (range)
TB Patient Kit	21 (25.3%)	12.93 (0–158)
RHZ 75/50/150 mg	14(16.9%)	5.22 (0–79)
RH 75/50 mg	33(39.8)	16.81 (0–128)
INH 300 mg	26 (31.3%)	18.30 (0–203)
INH100 mg	16(19.3%)	9.84 (0–240)
Ethambutol 100 mg	25(30.1%)	24.24 (0–194)

### Frequency of stock outs of first line anti-tuberculosis drugs

Taking the average number of stock outs, the most frequently stock out was observed for INH 300mg (Isoniazid) (0.66 with a maximum of 6 times of stock outs with a year) and followed by RH75/50 mg for which the mean frequency of 0.6 observed with maximum of 3 times of stock outs in the same period.

### Status of training of participants

74(89.2%) of the participants have been trained on Integrated Pharmaceutical Logistics System (IPLS) while only 14(16.87) of participants took anti-TB Drug supply and Management (DSM) training.

### Determinants for stock outs of first line anti-TB drugs

#### Information and communication technology (ICT) practice of the facility

As shown in Table 2, thirty-eight and twenty-six participants were agreeing and strongly agree respectively on the involvement of facility in the logistic social media group. Thirty-two and nineteen participants were agreeing and strongly about facility utilization of SMS for exchange of logistic information while twenty-one participant each agreed and strongly agreed on the utilization of internet for exchange of logistic information. Thirty-four and forty participants were agreeing and strongly agreed respectively for use of electronic Logistics Management Information System (LMIS) by the facility.

**Table 2:** Information and communication technology (ICT) practice of the Facility

Items	Strongly Disagree	Disagree	Neutral	Agree	Strongly Agree
Involvement of the facility in logistics social media group (Telegram, viber, WhatsApp)	9 (10.8%)	4(4.8%)	6(7.2%)	38(45.8%)	26 (31.3%)
Practice of the facility using SMS for exchange of logistics information	12(14.5%)	16(19.3%)	4(4.8%)	32(38.6%)	19(22.9%)
Use of internet by the facility for Exchanging logistics information	18(21.7%)	21(25.3%)	2(2.4%)	21(25.3%)	21(25.3%)
Use of electronic LMIS by the facility	4(4.8%)	3(3.36%)	2(2.4%)	34(41%)	40(48.2%)

#### Health system support activities at the health facility

As shown on Table 3, majority of respondents (forty-nine of them agree and twenty-five of them strongly agree) had positive response about supportive supervision provided for their facility while twenty-five and fifty-seven respondents agree and strongly agree respectively about the drug store are managed by academically qualified staff. Regarding the existence of in-service training related to pharmaceuticals logistics management, forty-five and twenty-seven of participants were agreeing and strongly agreeing respectively. Thirty-three and forty-four of the participants agreed and strongly agreed respectively about the reliability of logistic data which were sent to Ethiopian Pharmaceuticals Supply Agency (EPSA) While thirty-two and forty-seven of them respectively agreed and strongly agreed on timely submission of Report and request form (RRF) to EPSA.

**Table 3:** Respondents perception towards health System Support Activities at the Health Facility

Items	Strongly Disagree	Disagree	Neutral	Agree	Strongly Agree
The facility received supportive supervision	2(2.4%)	1(1.2%)	6(7.2%)	49(59%)	25(30.1%)
Drug Store are Managed by academically qualified staff	0	1(1.2%)	0	25(30.1%)	57(68.7%)
Existence of in-service training related to pharmaceuticals logistics management	4(48%)	6(7.2%)	1(1.2%)	45(54.2%)	27(32.5%)
Reliable logistics data sent to EPSA	2(2.4%)	0	4(4.8%)	33(39.8%)	44(53%)
Pharmaceuticals are ordered by the facility without any bureaucracy	3(3.6%)	11(13.3%)	3(3.6%)	38(45.8%)	28(33.7%)
RRF is timely submitted to EPSA	0	3(3.6%)	1(1.2%)	32(38.6%)	47(56.6%)

#### Performance of the supplying hub (EPSA)

As shown on Table 4, forty-one and twelve participants respectively agreed and strongly agreed about delayed supply of anti TB drug from EPSA while forty-eight and six respectively agreed and strongly agreed about ordered quantity of Anti-TB drugs reduced by EPSA. Thirty-six and twelve of participants agreed and strongly agreed respectively about stock out of Anti-TB drug in the previous year while thirty-eight and twelve of participants agreed and strongly agreed respectively on the short expiry date of Anti-TB drug supplied by EPSA.

**Table 4:** Respondents perception towards performance of the Supplying Hub (EPSA)

Items	Strongly Disagree	Disagree	Neutral	Agree	Strongly Agree
Delay of anti TB drug supply from EPSA	3(3.36%)	21(25.3%)	6(7.2%)	41(49.4%)	12(14.5%)
EPSA supplies anti TB drugs reducing quantity Ordered	8(9.6%)	16(19.3%)	5(6%)	48(57.8%)	6(7.2%)
There has been Stock out of anti TB Drugs at EPSA in the previous year	13(15.7%)	17(20.5%)	5(6%)	36(43.4%)	12(14.5%)
The Facility faces supply of short expiry anti TB drugs by EPSA	8(9.6%)	21(25.5%)	6(7.2%)	38(45.8%)	10(12%)

#### Findings from logistic regression analysis

As shown on Table 5, delayed supply of Anti-TB drug from EPSA significantly increased stock out of at least one anti-TB drug by 10 times (10.34(2.167–49.329) at the health facilities. Stock out of at least one anti-TB drug from health facilities significantly increase by 11 times when EPSA reduced the quantity of anti-TB drug ordered and increase by 6 times when there were stock out at EPSA.

**Table 5:** Logistic regression analysis

Variables	No	Yes	COR	AOR
Delay of anti TB drug supply from EPSA	Yes	53	39.167(10.866–141.173)	10.34(2.167–49.329) *
	No	30	1	**1**
EPSA supplies anti TB drugs reducing quantity Ordered	Yes	54	32.229(9.247–112.325)	11.452(2.183–60.079) *
	No	29	1	**1**
There has been Stock out of anti TB Drugs at EPSA in the previous year	Yes	48	5.700(2.159–15.049)	5.646(1.240–25.707) *
	No	35	1	**1**

#### Findings from key informants in in-depth interview

Stock out of TB patient Kit and reasons for stock out:

- ➢ “TB patient Kit was stocked for about 3 months and the reason mainly was delay in shipment from the external supplier compony and I think delay in payment from the country also contributed to the shipment delay” Key Informant 1 (KI 1)
- ➢ Even though all anti TB drugs should always be available at health facilities most of the health facilities were stocked out for pediatrics formulations and not manage it at all.
- ➢ “Tracer drugs including anti TB drugs should always be available at health facilities but there is knowledge gap at health facilities on identifying which anti TB Drugs to request especially new formulations such as new pediatrics formulations, even sometimes requests are made on already phased out formulations such as E400 mg and also newly introduced items were not captured by request and request formats ,thus some still use the old format and mistakenly request already phased out drugs and drop the one should be requested hence, most of the time stock outs reported were artificial” .KI 2

#### RRF is not timely submitted to EPSA

Regarding this pediatrics formulations another key informant said:

- ➢ “When you request responsible people at health facilities on availability of Pediatrics anti TB formulations, they mainly tell you that these items are stocked out, and they blame the supplying hub for this but, most of the time early requests on these items were not being placed because the number of pediatrics patients are small and arrive for treatment very intermittently. Hence, this created negligence at health facilities and attention was not being paid, and they tend to request these drugs after patient arrive to their facility, or they refer the patient to another facility which stocked these drugs claiming they don’t have the drug to start the treatment”. KI 3
- ➢ “Currently, we don’t have complete item for treating pediatrics patients, this is because there are not pediatrics cases at our facility but when a patient comes, we borrow from a nearby health facility and no patient has been made to leave without treatment.” KI 4

## Discussion

The finding from this research revealed that 52(62.7%) of health facilities encountered stock out for at least one of these drugs during the past 1 year. A national survey in south Africa showed stocked out rate during 2010/11 were 14.2% and 10.7% during 2012/13. Vhembe district had the highest stock out (41.1) during 2012/13 while A Nzo district (40.1%) during 2010/11 [19]. A recent study were also revealed 16.52% stock out of TB drug in northern cape [7]. The difference between stock out rate among different countries may be because of different approach of TB programs and time of study.

The finding from this study were much higher on the rate of stock out of TB patient kit 21(25.3% ) and Isoniazid 300 mg 26(31.3%) than the study conducted on 2017 7% stock out for TB Patient Kit and 17.5% for Isoniazid 300 mg while showed lower stock out of pediatrics formulation [RH75/50 mg (33(39.8%)) and RHZ75/50 (14(16.9%)] than RH75/50 mg and R HZ75/50; 57 % and 82.5% respectively in the same study [10]. The higher level of stock out found by Mekonen in 2017 might be due to the transition to newer pediatrics formulations at the same period and the old formulations were under phaseout stage and due to transition time, most of the facilities encountered stock outs until the supplying hub delivers these drugs for all the health facilities [10].

Even if there was significant level of stock out, it is important to note that facility specific stockout does not necessarily imply that a patient does not receive treatment as health facilities have a practice of borrowing from nearby health facilities once patient comes and try to manage the patient [20].

There were different previous studies from other countries who have got the same result pertaining to supplier performance such as restock delay [4, 21] stock out at supplying hub [4, 22].

Stock out of medicine may have high impact and consequence on patients such as patients left the facility without medicine or provided with an incomplete regimen [23], risk of treatment interruption or discontinuation, catastrophic expenditure [24, 25] treatment failure and drug resistance and ultimately increased risk of illness and death [26, 27].

The major limitation of this study is related to the data source used for collection of the stock out data where secondary data sources, mainly bin cards were used and sometimes it was difficult to get updated bin cards which was resolved by triangulating data from paper based, electronic bin cards and further requesting the drug store managers.

## Conclusion

Above median of public health facilities encountered stock out of first line anti-TB drug in Addis Ababa. Delayed supply of anti TB drug from EPSA, reduction of delivery quantity of anti TB drugs by EPSA and stocked out of anti TB Drugs at EPSA were significant determinate factor of stocked out of first line anti-TB drug from facilities.

EPSA and other responsible bodies shall work collaboratively to improve their service and ensure availability of adequate amount of Anti TB drug in health facilities.

## Supplementary Information

**Additional file 1.**

## Data Availability

All data generated or analyzed during this study are included in this published article.

## Abbreviations

DSM: Drug Supply & Management
EFDA: Ethiopian Food and Drug Authority
EPSA: Ethiopian Pharmaceuticals Supply Agency
ICT: Information communication technology
IPLS: Integrated Pharmaceutical Logistics System
KI: Key Informant
LIAT: Logistics Indicators Assessment Tool
LMIS: Logistics Management Information System
LSAT: Logistics System Assessment Tool
RRF: Report and request form
SMS: Short message service
TB: Tuberculosis
