# Supplementary material for: Determinants of stock-outs of first line anti-tuberculosis drugs: the case of public health facilities of Addis Ababa city administration health bureau, Addis Ababa, Ethiopia
Source: BMC Health Serv Res. 2022 Aug 17;22:1047. doi: 10.1186/s12913-022-08430-3 (PMC9381400; doi:10.1186/s12913-022-08430-3)
Supplement: Supplementary file 1 — Additional file 1. [file 12913_2022_8430_MOESM1_ESM.docx]

**ANNEX 1: DATA COLLECTION TOOLS**

1. CONSENT AND INFORMATION SHEET

Dear Participant, my name is -------------. I am going to conduct a study and collect data on determinants for stock outs of anti TB drugs at public health facilities of Addis Ababa. The objective of the study is to assess the levels of anti TB drug stock outs from the previous 1 year to date and the determinants for this stock outs. The information you provide will be used to improve availability of anti TB drugs thereby contribute to improved care of TB patients and prevention of drug resistant TB. The study will identify gaps and determinants for stock out of anti TB drugs and provide recommendations for proper interventions by the concerned bodies. Your participation in filling the questionnaires and every aspect of the study is completely voluntary. Your name will not be written on any of the instruments or be kept in any other records and all information that you give me will be kept confidential and you are free to withdraw your consent and to discontinue participation at any time without any consequence. But since your sincere response is very crucial, I hope that you are going to participate in the study.

Thank you in advance for your cooperation!

N.B: If you want to request additional information about the study you can contact the principal investigator for the study.

Tel: --------------------------------and/ or email: -----------------

You are now being asked if you have read the information above and if you are willing to participate in the study. We would greatly appreciate your truthful and keen participation in responding to this questionnaire.

Yes, I agree _____________ No, I don’t agree__________

Participants Signature______________

Thank you!

**2. QUESTIONNAIRE**

Research title: Determinants of stock-outs of first line anti-tuberculosis drugs: the case of public health facilities of Addis Ababa city administration health bureau, Addis Ababa, Ethiopia.

Questionnaire for Drug Store Managers

##### Part I: Background Information

1. Gender Female


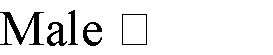

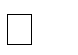


1. Profession:
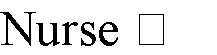


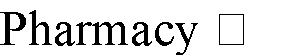

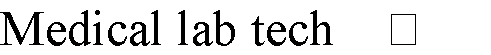

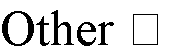


1. Age: __________________
2. What is your level of education? Diploma


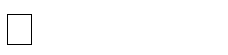

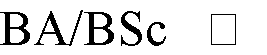


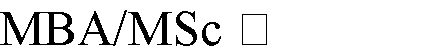
 Others (specify) ………

1. How many years (months) have you been working in this capacity? ------------------- 6. Have you ever received any training on integrated pharmaceuticals logistics system (IPLS)?


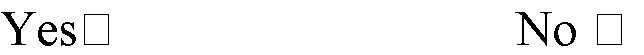


7. Have you ever received any training related to TB DSM?


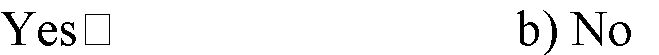


Part II: General Questions

Please indicate your level of agreement on the statements for each question using the following rating scale by putting “X”

Key: 1= Strongly Disagree, 2= Disagree, 3= Neutral, 4 = Agree, 5 = Strongly Agree

| **Information and Communication Technology (ICT) Use Practice of the facility** | | | | | |  |
| --- | --- | --- | --- | --- | --- | --- |
| SN | Items | Five scale rating | | | |  |
|  |  | Strongly  Disagree  = 1 | Disagree  = 2 | Neutral  = 3 | Agree  = 4 | Strongly  Agree  =5 |
| 1 | The facility involves in pharmaceutical logistics social media group (Viber, Telegram) for exchanging logistics information |  |  |  |  |  |
| 2 | The use of SMS messaging and phone call for exchange of logistics data with stakeholders involved in pharmaceutical logistics management is practiced in the facility |  |  |  |  |  |
| 3 | The use of an internet to exchange pharmaceutical logistics data with stakeholders involved in pharmaceuticals logistics management is practiced in the facility |  |  |  |  |  |
| 4 | The facility utilizes electronic LMIS |  |  |  |  |  |
| **Health System Support Activities at the Health Facility** | | | | | |  |
| SN | Questions | Four scale rating | | | |  |
|  |  | Strongly  Disagree  = 1 | Disagree  = 2 | Neutral  = 3 | Agree  = 4 | Strongly  Agree  =5 |
| 1 | The facility received supportive  Supervision from higher level  (RHB, EPSA, FMOH etc) in the |  |  |  |  |  |
|  | previous one year |  |  |  |  |  |
| 2 | Pharmaceuticals in the drug store are managed by academically qualified staff (Trained Druggist or Pharmacist) |  |  |  |  |  |
| 3 | There exists in-service training related to pharmaceuticals logistics management for professionals handling pharmaceuticals |  |  |  |  |  |
| 4 | Reliable logistics data is sent from the facility to EPSA is (as verified from last year RRFs cross checked with bin/stock card) |  |  |  |  |  |
| 5 | Pharmaceuticals are ordered by the  health facility without any bureaucracy |  |  |  |  |  |
| 6 | The facility RRF is timely submitted to EPSA (as verified from date of submission for last year RRF) |  |  |  |  |  |
| **Performance of the Supplying Hub (EPSA)** | | | | | | |
| S.N | Questions | Five scale rating | | | | |
|  |  | Strongly  Disagree  = 1 | Disagree  = 2 | Neutral  = 3 | Agree  = 4 | Strongly  Agree  =5 |
| 1 | There exists delay in delivery of anti-TB drugs from EPSA |  |  |  |  |  |
| 2 | EPSA Reduces quantity of anti-TB drugs ordered by the facility (it supplies reducing from ordered quantity) |  |  |  |  |  |
| 3 | There has been Stock out of antiTB drugs at EPSA in the previous year |  |  |  |  |  |
| 4 | The facility faced Supply of short expiry anti TB drugs by EPSA |  |  |  |  |  |

##### 3. OBSERVATIONAL CHECKLIST TO ASSESS LEVEL OF STOCK OUT OF ANTI TB DRUGS

Fill in the columns based on inspection on inventory cards / Bin or Stock cards

| S  N | Anti TB drug | stock outs within the study period  (March 20, 2019, to  March 20, 2020  (Yes / No): | If stock out, number of stock outs (Frequency /how many times) within the study period | Total DOS (previous one year up to the day of the study |
| --- | --- | --- | --- | --- |
| 1 | TB patient Kit |  |  |  |
| 2 | RHZ (75/50/150 mg) |  |  |  |
| 3 | RH (75/50 mg) |  |  |  |
| 4 | INH 300 mg |  |  |  |
| 5 | INH 100 mg |  |  |  |
| 6 | Ethambutol 100 mg |  |  |  |

##### INTERVIEW GUIDE

Guidelines for semi
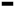
 structured interviews with Pharmacy heads, Respondents from RHB and EPSA. (To be modified for each of the stakeholders during the interview)

Part I: Background Information


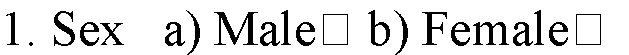

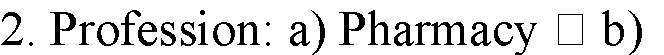

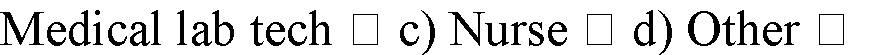


1. Age: __________________
2. What is your level of education? a) Diploma


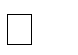

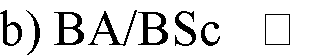

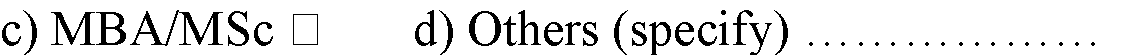


1. What is your position? ----------------------------
2. How many years (months) have you been working in this capacity? -------------------
3. Have you ever received any training in integrated pharmaceuticals logistics system (IPLS) ?


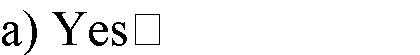


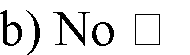


If yes, When? ---------------------------------

1. Have you ever received any training related to TB DSM?


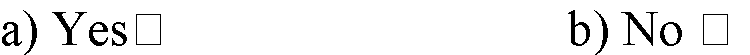


If yes, When? ---------------------------------

**Part II: General Questions**

1. Now I would like you to think about the past 1 year. Have you ever faced SO of anti TB drugs in the previous year (from March 20, 2019, to date)?
2. Which anti TB drugs have been stocked out in the previous year including today?
3. What do you think are the determinants for mentioned Stock outs? (Need for further probing in associated with the variables etc)
   - The use of information and communication technology (ICT)
   - Health system Support
   - Performance of EPSA

1. In your opinion, what can be done to address these identified determinants and improve availability of anti TB drugs in your facility?

Is there anything else you would like to tell us or ask us?

Thank you very much for your participation in this interview.
